# The state of art of neutrophil extracellular traps in protozoan and helminthic infections

**DOI:** 10.1042/BSR20180916

**Published:** 2019-01-11

**Authors:** César Díaz-Godínez, Julio C. Carrero

**Affiliations:** Department of Immunology, Instituto de Investigaciones Biomédicas, Universidad Nacional Autónoma de México, 04510, México D.F., México

**Keywords:** helminths, neutrophils, NETosis, NETs, parasites, protozoa

## Abstract

Neutrophil extracellular traps (NETs) are DNA fibers associated with histones, enzymes from neutrophil granules and anti-microbial peptides. NETs are released in a process denominated NETosis, which involves sequential steps that culminate with the DNA extrusion. NETosis has been described as a new mechanism of innate immunity related to defense against different pathogens. The initial studies of NETs were carried out with bacteria and fungi, but currently a large variety of microorganisms capable of inducing NETs have been described including protozoan and helminth parasites. Nevertheless, we have little knowledge about how NETosis process is carried out in response to the parasites, and about its implication in the resolution of this kind of disease. In the best case, the NETs entrap and kill parasites *in vitro*, but in others, immobilize the parasites without affecting their viability. Moreover, insufficient studies on the NETs in animal models of infections that would help to define their role, and the association of NETs with chronic inflammatory pathologies such as those occurring in several parasitic infections have left open the possibility of NETs contributing to pathology instead of protection. In this review, we focus on the reported mechanisms that lead to NET release by protozoan and helminth parasites and the evidence that support the role of NETosis in the resolution or pathogenesis of parasitic diseases.

## Introduction

Parasitic diseases are a relevant public health problem of chronic diseases due to the high capacity of the parasites to evade immune responses [1]. Parasites greatly contribute to the global burden of infectious diseases in the developing world, mainly in tropical and subtropical communities. According to the World Health Organization (WHO) diarrheal diseases are the most frequent illness in humans, and several species of enteric parasites are partially responsible for them [2]. Drinking water contaminated with feces carrying parasite cysts or eggs can be the source of infection of parasitic diseases caused by protozoans such as amoebiasis, giardiasis, toxoplasmosis, cryptosporidiosis, and blastocystiosis or by helminths such as ascariasis and trichuriasis [3,4]. Despite the advances in the diagnosis and treatment of these diseases, the number of outbreak cases of water-borne parasitic diseases seems to have increased worldwide in recent years [5]. The reasons are unknown, but it may have to do in part with the climate changes [6] and the deterioration of drinking water sources in the communities [5]. The impact of the parasitic diseases is more frequent in developing countries but they also cause significant illness in the developed world [7]. In addition, the parasitic diseases represent great economic losses derived from the costs of diagnosis, treatment, and disability [8]. All the above indicates that parasitic infections remain one of the priorities within the scope of human and animal health and that the study of the strategies related to their control, including the host immune response, is of paramount importance in the field of parasitology.

Neutrophils are the most abundant leukocytes in the peripheral blood of mammals. These cells are rapidly recruited at the sites of infection where they form the first immunological barrier against pathogens using different combat strategies including phagocytosis [9–12], degranulation [13–16], and neutrophil extracellular traps (NETs) formation. NETs are nuclear DNA fibers associated with histones, enzymes of cytoplasmic granules and anti-microbial peptides, which are released into the extracellular space [17]. Different mechanisms of anti-pathogen action have been attributed to NETs including microorganism immobilization, degradation of virulence factors, and killing of bacteria, fungi, and parasites [17–20]. NETs are released in a process denominated NETosis that originally was described as a new mechanism of cell death [21]; nevertheless, cases of vital NETosis have been reported in which neutrophils do not lose their viability because they release mitochondrial DNA instead of nuclear DNA [22].

Initially, NETosis was extensively characterized using phorbol miristate acetate (PMA) as stimulus. It was shown that PMA activates protein kinase C (PKC) and Raf/MEK/ERK signaling pathway [23,24]. Posteriorly, NADPH oxidase complex is assembled, which generates reactive oxygen species (ROS) [22]. ROS induce an oxidative dissociation of neutrophil elastase (NE) from azurosomes present in azurofilic granules; this enzyme is translocated to the nuclei where it cleaves histones causing DNA decondensation [25] that is enhanced on the association of myeloperoxidase (MPO) to chromatin [26]. In the next step, internal membranes are dissociated, DNA associated with proteins derived from the granules, and, finally, cytoplasmic membrane disrupts releasing NETs [21]. NETosis process induced by calcium ionophores is independent of ROS generated by NADPH oxidase but it depends on peptidyl arginine deiminase 4 (PAD4) [27,28], a nuclear enzyme that catalyzes the conversion of positively charged arginine residues present in histones into non-charged citrulline residues [29]. PAD4 is activated by an increase in calcium influx and promotes chromatin decondensation [27]. Recently, it was described that ROS from mitochondria are required for NETosis induced by calcium ionophores [30]; nevertheless, the role of mitochondrial ROS remains unknown.

Both oxidative and non-oxidative responses have been detected during the interaction of neutrophils with parasites. It has been reported that oxidative burst is triggered in neutrophils by the lysates, excretory/secretory products and plasma membrane components from *Trichomonas vaginalis* [31], *Leishmania amazonensis* and *Leishmania braziliensis* amastigotes [32], and *Entamoeba histolytica* trophozoites [33], as well as by whole extracts, scolex, and membranes from *Taenia solium* metacestode [34], to mention some examples. Degranulation as a part of the non-oxidative mechanism used by the neutrophils in their struggle against parasites has been reported in cells stimulated with *Leishmania* [32]. In addition, phagocytosis has been effective in the control of small-size parasites as *Trypanosoma cruzi* and some species of *Leishmania* [35–37]; nevertheless, this mechanism was not effective against large parasites such as helminths since they cannot be engulfed. In this regard, it has been suggested that NETosis plays an active role in the defense against helminth parasites because they are entrapped by NETs preventing dissemination. Even though some small parasites are phagocytized by neutrophils, these immune cells can also release NETs that affect the viability of parasites and prevent their invasion.

The decision of neutrophils to generate NETs instead of phagocytosis is another area of intense study. The decision seems to be the result of the combination of multiple signals including the adhesive, metabolic, and activation conditions of the cells, the stimuli from the environment, and, very importantly, the size and signals coming from the stimulating particle (alive or inert; reviewed in [38]). Regarding the size of the stimulating particle, it has been suggested that large particles such as parasites would induce the formation of NETs, while small particles such as bacteria and viruses should be eliminated by phagocytosis [39,40]. It is well known, however, that both viruses and bacteria can induce NETs formation under certain conditions, so other aspects such as the signals derived from the particle (e.g., pathogenicity and/or virulence signals) are also important for the outcome under physiological conditions [41,42]. Noteworthy, whatever the decision made by the neutrophils, phagocytosis and NETosis seem mutually exclusive. The reasoning behind this has to do with the availability of the enzymes NE and MPO to perform functions in the nucleus or in the cytosol and phagolysosomes of neutrophils. During phagocytosis, both enzymes are recruited to the phagolysosomes that keep them away from the nucleus whereas during NETosis the enzymes are released from the azurosomes into the cytosol to degrade the actin cytoskeleton and then be translocated to the nucleus where they participate in chromatin decondensation. The degradation of the cytoskeleton and the sequestration of NE and MPO into the nucleus, keeping them away from the endocytic pathway, would impede the function of phagocytosis (reviewed in [38]). Despite all this, the physiological role of the NETs during parasitic infections remains unknown and is the subject of intense study.

Here, we provide an update of what is known regarding NETosis induced by parasites, the mechanisms involved in its triggering, and the outcome of the exposition of parasites to NETs in terms of their viability and infectivity. We also discuss the implications that manipulation of NETs may add to the control of parasitic diseases.

## Protozoan parasites: an overview

Infections by protozoan parasites represent a high risk to public health, as they are responsible for many outbreaks worldwide [43,44]. Protozoan parasites are cited as one of the main causes of 1.7 billion annual cases of diarrhea contributing significantly to the 842000 deaths associated; these data place them as the second leading cause of death in children under 5 years old [45,46]. According to the Global Burden of Disease Study 2016, the neglected tropical diseases and malaria were responsible for 843600 deaths, malaria alone being responsible for 85% of them [47]. Other protozoan parasitic diseases such as leishmaniasis, Chagas, and African trypanosomiasis were responsible for 13700, 7100, and 2300 deaths, respectively, in 2016 [47]. On the other hand, amoebiasis that caused approximately 55500 deaths worldwide in 2010 is considered as the second leading cause of death by protozoan parasites [48].

During the infection with protozoan parasites, the innate immune cells such as macrophages and neutrophils contribute crucially to the host immune response. Together with dendritic cells, Natural killer cells, and natural killer T cells, macrophages and neutrophils promote the development of the pro-inflammatory Th1-profile adaptive response that controls the parasite and therefore the disease. Neutrophils are the first immune cells recruited to the infection sites, so constituting the first line of defense of the cellular innate immunity [49]. The early recruitment of neutrophils to the site of tissue invasion usually contributes to reduce the initial burden of protozoan parasites. As mentioned above, effector activities of neutrophils against protozoan parasites include degranulation, phagocytosis (when the parasite is enough small to be phagocyted), oxidative burst, and, ultimately, the formation of NETs.

## Protozoan parasites and NETs

### *Leishmania* spp.

Parasites of genera *Leishmania* actually have a relevant prevalence in endemic regions. According to WHO, more than 5000 cases of cutaneous leishmaniasis were reported in 2015 in endemic countries of South America (Peru, Colombia, and Brazil), Asia (Syria, Iraq, Iran, Afghanistan, and Pakistan), and Africa (Algeria and Tunisia), while more than 1000 cases of visceral leishmaniasis were reported during the same year in Brazil, Sudan, Uganda, Ethiopia, and Somalia [50]. This parasite is transmitted by female sandflies after a blood meal, injecting the promastigotes (infective stage) in the mammal host, which are phagocyted by resident host macrophages within which they transform into amastigotes [51].

Different groups have described *in vivo* and *in vitro* interaction of neutrophils with *Leishmania* species. Neutrophils are rapidly recruited at the infection sites after inoculation of BALB/c mice with *L. amazonensis*, reaching the highest infiltration levels after 18 h; when the neutrophils are depleted, a major progression of the lesions and an increase of parasite burden take place [52]. Neutrophils can phagocyte amastigote and promastigote forms of *L. amazonensis*, resulting in their activation denoted by an increase in CD11b expression and ROS generation by NADPH oxidase. This triggering of neutrophils leads to the parasite killing; nevertheless, amastigotes seem to be more resistant to the anti-parasitic action of neutrophils compared with promastigotes [37]. Neutrophils were also able to phagocyte *L. major* promastigotes through the CR3 complement receptor, so the phagocytosis increased when the parasites were opsonized. Interestingly, only under this situation promastigotes triggered neutrophils activation [53].

Guimaraes-Costa et al. [54] were the first to describe NETosis induced by *Leishmania*; the authors reported that *L. amazonensis, L. chagasi*, and *L. major* promastigotes induced NETs release in naïve neutrophils and detected the presence of histones and NE in these structures. It was observed using immunofluorescence and scanning electron microscopy that promastigotes were trapped in NETs and showed morphological alterations, suggesting loss of their viability. Interestingly, either amastigotes or promastigotes were able to induce NETosis in non-activated neutrophils, indicating that the parasite stage is not a determinant factor to induce NET release. This group considered that histones present in the NETs were the effector molecules because the use of anti-histone H2 antibodies increased the survival of parasites and purified histone H2 reduced the viability of promastigotes. Noteworthy, the use of lipopetidoglycan (LPG) from *Leishmania* also induced NETosis in a dose-dependent manner.

Posteriorly, it was reported that *L. donovani* promastigotes also induced NETosis in a dose-dependent manner and that no differences were observed when either opsonized or non-opsonized promastigotes were tested [55]. In this study, NETosis occurred rapidly 10 min after stimulation of neutrophils and required a close contact between neutrophils and promastigotes. It is important to mention that contrary to the previous observation, LPG present on the surface of *L. donovani* was not necessary to induce NETosis since mutant promastigotes lacking LPG did not show reduced capacity to induce NETs as well as LPG-coated zymozan failed to promote NET release. Nevertheless, LPG showed protective activity against the anti-parasitic effect of NETs since mutant promastigotes lacking LPG showed less survival percentage upon the action of NETs as compared with wild-type promastigotes. The authors also gave a first approximation to understand the mechanism involved in the NET release induced by *L. donavani*, probing that ROS generated by NADPH oxidase were not necessary for this NETosis process [55].

A work aimed to understand the mechanisms implicated in the NETosis by *L. amazonensis* was performed by Rochael et al. [56]. They demonstrated that inhibition of NE and PAD4 using MeOSuAAPV-CMK and chloroamidine, respectively, reduced the amount of NETs released after exposure of neutrophils to promastigotes; in contrast, MPO inhibition did not affect the NETosis. In this study, it was demonstrated that *L. amazonensis* promastigotes caused an increase of H2O2 levels in neutrophils and that ROS were generated by NADPH oxidase and mitochondrial respiratory chain. Nevertheless, only ROS derived from NADPH oxidase complex seemed to be necessary for the NETosis process since inhibition of other ROS sources such as xanthine oxidase did not affect the release of NETs induced by *L. amazonensis*, while nitric oxide synthase inhibition reduced the NET amounts. An early/rapid NETosis induced by promastigotes (only 10 min after stimulation) dependent on NE activity but independent of NADPH oxidase-derived ROS and PAD4 activity was also described in this work indicating that different pathways that converge in NET release could be triggered by the same stimulus [56].

**Figure 1:**
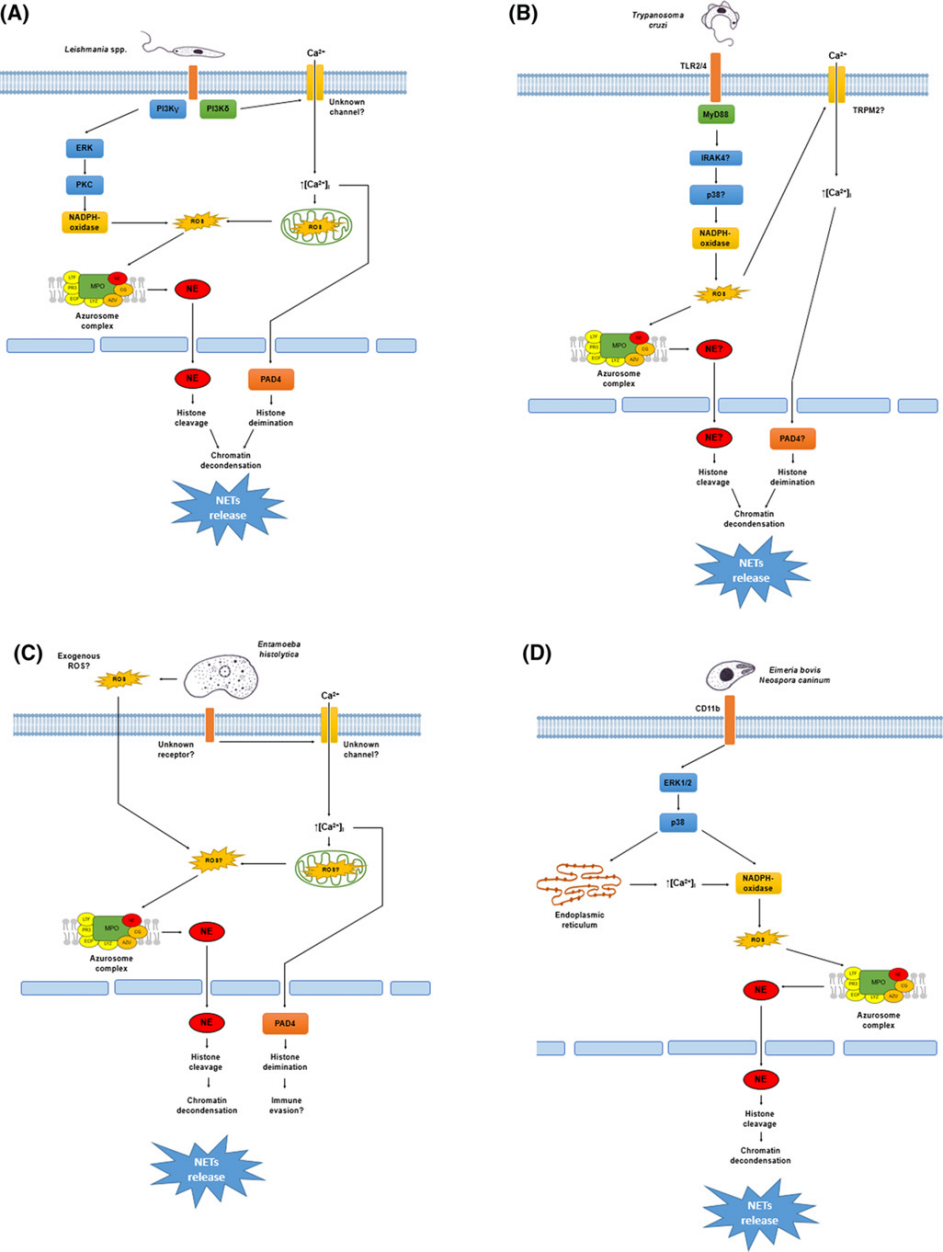
NETosis mechanisms triggered by protozoan parasites (**A**) *Leishmania* spp. promastigotes activate the PI3K signal pathway. PI3Kγ isoform leads to activation of the ERK pathway that phosphorylates PKC promoting the NADPH oxidase assemble. The NADPH oxidase generates ROS causing oxidative dissociation of the NE from the azurosome complex present in the membrane of azurophilic granules. NE is translocated to the nucleus where this enzyme cleaves the histones promoting chromatin decondensation and NET release. The PI3Kδ isoform probably causes calcium influx through an unknown membrane channel promoting ROS generation from the mitochondrial respiratory chain. ROS lead to the nuclear translocation of NE as mentioned above, or the influx of calcium activates the PAD4 causing histone citrullination and chromatin decondensation. (**B**) *Trypanosoma cruzi* trypomastigotes are sensed by the TLR2/4 receptor complex leading to NADPH oxidase assemble via the MyD88/IRAK4/p38 pathway. ROS promote NE translocation to the nucleus from the azurosome complex or activate the TRPM2 channels leading to calcium influx, which activates PAD4. Both pathways converge in the chromatin decondensation and NET release by histone cleavage or histone citrullination, respectively. (**C**) *Entamoeba histolytica* trophozoites trigger NETosis dependent on NE activity and extracellular calcium influx but independent of NADPH oxidase-derived ROS. It is likely that other ROS sources could be implicated in NETosis induced by amoebas since exogenous ROS from trophozoites or ROS from the mitochondrial respiratory chain can promote NE translocation, chromatin decondensation, and NET release as described elsewhere. In contrast, the trophozoites cause calcium influx that activates PAD4 promoting protein citrullination. Since PAD4 activity is not necessary for NETs release by amoebas, protein citrullination could be a mechanism of the parasite to evade the anti-microbial action of NETs. (**D**) The Apicomplexa parasites *Eimeria bovis* and *Neospora caninum* are sensed by neutrophils throughout the CD11b receptor, which in turn activates the ERK1/2 signaling pathway. The ERK pathway leads to calcium mobilization from the storages operated calcium entry (SOCE) that promotes NADPH oxidase activation or directly promotes NADPH oxidase assemble by phosphorylation. ROS are generated from this complex and the NE translocation takes place to decondense chromatin for release of NETs.

The molecular pathways involved in NETosis induced by *L. amazonensis* were studied by DeSouza-Vieira et al. [57]. They demonstrated that there are two pathways implicated in the NETosis by this parasite: one dependent, and another independent of ROS generated by NADPH oxidase. The ROS-dependent pathway implicates the activation of PI3K-γ, which activates ERK via MAPK; posteriorly, ERK activates PKC and ROS are generated by NADPH oxidase. ROS generation during ROS-dependent NETosis probably leads to the NE dissociation from the azurosomes and promotes histone cleavage that decondense DNA, as have been reported [25,26]. On the other hand, ROS-independent NETosis triggered by this parasite activates PI3K-δ that leads to an increase in calcium mobilization. No more inhibitors were tested to determine downstream components; nevertheless, NE activity was required to extrude DNA [57]. The ROS-independent NETosis that leads to an increase in cytoplasmic calcium levels could be able to activate PAD4 enzyme to decondense DNA [27]; PAD4 inhibition, however, did not affect this NETosis. Since reports exist indicating that mitochondria-derived ROS are necessary for NADPH oxidase-independent NETosis processes induced by calcium ionophores [30], it is likely that the mitochondria is the source of the ROS necessary for the induction of NETs by *L. amazonensis*. In addition, it is reported that the increase in calcium mobilization produced an increase of mitochondrial ROS in other cell types [58]. In this sense, ROS originated from mitochondria could dissociate NE to promote chromatin decondensation and release NETs (Figure 1A).

### Trypanosoma cruzi

Chagas disease is a parasitosis with high prevalence in Mexico and Central and South America. According to the WHO, 8 million people are infected with *T. cruzi* worldwide causing more than 10,000 deaths per year [59]. *T. cruzi* is transmitted by different species of triatomine insects including *Triatoma, Rhodnius*, and *Panstrongylus*. After a blood meal, the insect vector releases in its feces the metacyclic trypomastigotes that enter the host through the lesions caused by the triatomine. Oral infection through ingestion of fruits and food contaminated with insect’s feces and via the transplacental route has also been reported. Inside the host, trypomastigotes invade adjacent cells and differentiate into amastigote forms. Amastigotes divide by binary fission and differentiate into trypomastigotes that are released into blood stream. Trypomastigotes can invade different organs as heart, colon, and esophagus, causing tissue damage with chronic manifestations [60,61].

Different groups have studied interaction between neutrophils and *T. cruzi*. It is described that neutrophils are able to phagocyte trypomastigote and amastigote forms of *T. cruzi* [36,62] and that neutrophils can kill the parasites in the phagolysosome vacuole through a mechanism dependent on the generation of hydrogen peroxide since neutrophils cultured with the parasite in the presence of catalase showed reduced killing efficiency [35,36]. In another study, neutrophil-depleted BALB/c mice showed increased parasitemia and less survival to acute infection, suggesting a protective role of neutrophils against *T. cruzi* [63]. In contrast, neutrophils apparently contribute to the pathology associated with trypanosomiasis because cardiac myoblasts are damaged when co-cultured with neutrophils and amastigotes, a trypanosomal state that cannot infect myoblast. The damage is likely mediated by secretory products from neutrophils since supernatants from neutrophil-parasite co-cultures also promote the detachment of myoblasts [64].

It has been described that *T. cruzi* trypomastigotes and their soluble extracts induce NETosis in a dose-dependent manner [65]. This mechanism appears to be triggered by sensing via TLR2/4 and involves the generation of ROS and histone citrullination. Actually, it is poorly understood how the TLR signaling pathway leads to NETosis; nevertheless, evidence exist that stimulation of neutrophils with LPS via TLR4 can activate NADPH oxidase by the phosphorylation of p47phox in an MyD88-IRAK4-p38MAPK-dependent manner with the subsequent ROS generation (reviewed in [66]). It can explain why NETosis triggered by *T. cruzi* is dependent on ROS from NADPH oxidase; it has been described, however, that phagocytosis inhibits NETosis since the phagolysosomes sequester the NE preventing its translocation to the nucleus and chromatin decondensation [39]. In this regard, it has been described that neutrophils possess TRPM2 channels, members of the transient receptor potential family, that can be activated by hydrogen peroxide produced via the respiratory burst under physiological conditions (reviewed in [67]). This calcium influx through the TRPM2 channels might trigger the activation of PAD4 that decondenses the chromatin. It is unknown if this channel is activated during *T. cruzi*–neutrophil interaction, however, it could explain why neutrophils enter NETosis even when they are able to phagocyte trypanosomes.

NETs can reduce the infectivity of *T. cruzi* trypomastigotes [65] but it remains unknown why it is possible. Different NET components, such as NE, cathepsin G, and Proteinase 3, possess proteolytic activity and these enzymes can likely act over the surface molecules of *T. cruzi* necessary for infection, degrade them in a similar manner as it occurs with other microorganisms [17], and block the entrance of the parasite into the cells (Figure 1B). Nevertheless, more studies are necessaries to confirm this theory.

### Entamoeba histolytica

*E. histolytic*a is an intestinal parasite with high prevalence in developing countries [68–70] that is responsible for intestinal amoebiasis and amoebic liver abscess. It is transmitted by the ingestion of mature cysts from food or water contaminated with stools. The excystation process takes place in the small intestine and the released trophozoites migrate to the large intestine where they divide by binary fission and penetrate the intestinal mucosa [71]. Hosts may suffer an asymptomatic infection or develop amoebic dysentery. In some cases, trophozoites can reach the blood stream and establish in other organs, mainly the liver, causing amoebic liver abscesses [72].

Neutrophils are rapidly recruited at *E. histolytica* infection sites and seem to participate in the protection from amoeba since CBA mice, which are partially resistant to intestinal amoebiasis, suffer a greater percentage of infection and an increase in the cecum thickness compared with the control group in the conditions when neutrophils are depleted [73]. *In vitro* experiments showed that trophozoites induce respiratory burst and apoptosis in human neutrophils [33,74]. Nevertheless, the exact role of neutrophils in this parasitic disease remains unknown.

Recently, it was observed that *E. histolytica* trophozoites induce NET release in human neutrophils, however, these NETs were incapable to kill trophozoites and the treatment of amoebas with PMA-induced NETs did not affect the capacity of trophozoites to form amoebic liver abscesses in a hamster model and neither reduced engulfment of neutrophil by trophozoites [75]. Different molecules with amoebicidal effect have been described to form part of the amoebas-induced NETs, including the anti-microbial peptide LL-37 and other defensins, lactoferrins, and enzymes such as MPO and NE [17,75,76]. Though anti-microbial peptides possess a characterized amoebicidal effect [77,78], recent studies indicate that association of these peptides with DNA significantly reduced their anti-microbial potential due to the negative charge of DNA that neutralizes the positive charges present in the peptides that are necessary for their microbicidal effect [79,80]. In addition, the particularly high levels of cholesterol in the cytoplasmic membrane of *E. histolytica* trophozoites confer them protection against cationic peptides in a similar manner as described for high membranal levels of cholesterol protecting amoebas against their own amoebopore [81]. This could also explain why histones, which effectively kill other protozoan parasites [82], do not reduce viability of amoebas.

Our working group has recently studied the molecular pathways implicated in the NETosis induced by *E. histolytica*. We found that NETosis triggered by trophozoites depends on serine proteases activity and extracellular calcium and requires translocation of NE to nucleus. On the other hand, it also depends on ROS generated by NADPH oxidase, as well as on PAD4 activity and Raf/MEK/ERK signaling pathway [83,84]. Interestingly, NE translocation took place independently of NADPH oxidase activity, despite previous reports indicate that the dissociation of NADPH oxidase from the azurosome depends on ROS [25]. Nevertheless, other ROS sources such as mitochondrial respiratory chain or ROS from the NETs-stimulating cells have not been discarded since NETosis induced by calcium ionophores is dependent on ROS from mitochondria [30] and NETosis induced by *Candida albicans* in neutrophils from chronic granulomatous disease patients (that cannot form ROS) is dependent on ROS from the fungi [85]. It is to notice that trophozoites induced protein citrullination but PAD4 activity was not necessary for NET release. The significance of the citrullination of neutrophil proteins induced by the amoeba during the NETosis process that does not require PAD4 is unknown, but it has been suggested that citrullination of NET components by *E. histolytica* trophozoites could act as a mechanism to evade their anti-microbial action [86], which could help to explain why NETs failed to kill *E. histolytica* trophozoites.

In our laboratory, we have observed that other cell types, such as eosinophils and monocytes, are also able to form extracellular traps (ETs) in contact with viable *E. histolytica* trophozoites and that trophozoites of the non-pathogenic *E. dispar* did not trigger NETosis, suggesting a relationship between pathogenicity and NETs formation (unpublished data); however, the role of NETs and the ETs from other immune cells in the protection against this parasite remains unknown (Figure 1C).

### *Eimeria* spp.

Coccidiosis is a parasitic disease caused by apicomplexan protozoa of genera *Eimeria* that affects animals [87]. This disease causes diarrhea with or without blood and, in some cases, can result in the animal death. An animal is infected when it ingests sporulated oocysts that release sporozoites, which invade the intestinal epithelium. Sporozoites differentiate into meronts that divide by fission and develop into merozoites, which also invade the intestinal epithelium. Merozoites invade cells and develop into either macrogamonts or microgamonts. Macrogamonts develop into single macrogametes whereas microgamonts suffer multiple divisions resulting in numerous microgametes. Microgametes invade cells containing macrogametes and fertilization takes place resulting in oocysts formation [88].

The role of neutrophils in the protection against *Eimeria* is poorly studied; some evidence indicate that neutrophils may play an active role in the protection since neutrophil-depleted mice showed increased oocyst output after infection with *E. papillate* [89]. Nevertheless, the exact role of neutrophils in this infection is not yet clear.

The first description of NETosis induced by *E. bovis* was made by Behrendt et al. [90]. They found that *E. bovis* sporozoites trigger NETosis in bovine neutrophils much faster and stronger than PMA and that NETs were released in a time- and dose-dependent manner. Heat-inactivated sporozoytes and homogenates of active parasites failed to induce NETosis, suggesting that the viability of the parasite is indispensable for the induction NETs like it is observed with *E. histolytica* trophozoites [75,83]. This supports the notion that surface molecules present in pathogens are not the only stimuli necessary to induce NET release. The *Eimeria*-triggered process was dependent on ROS generated by NADPH oxidase, suggesting a classical NETosis pathway. In addition, sporozoytes that were co-cultured with neutrophils and underwent NETosis showed reduced infectivity in BUVEC cells. This effect was prevented using DNase, indicating the active role of NETs against this parasite. A more detailed work to study the mechanisms implicated in NETosis induced by *E. bovis* was performed by Muñoz-Caro et al. [91]. They described that CD11b receptor of neutrophils (an integrin component of complement receptor 3) was implicated in this NETosis since *E. bovis* sporozoytes induced its overexpression and the use of anti-CD11b receptor antibody reduced the NETs amount release by neutrophils co-cultured with the parasite. Like in other cases, NETosis required ROS from NADPH oxidase and the process was also dependent on calcium mobilization from store-operated calcium entry (SOCE), as well as on NE and MPO activities. *E. bovis*-induced NETosis was regulated by ERK1/2 and p38 MAPK pathways similar to what occurs with the cross-linking of FcγRIIIb receptor and PMA stimulation [24,92]. It is important to mention that merozoytes and oocyst stages of *E. bovis* also trigger NETosis, indicating that this process is not stage-specific. Moreover, not only bovine, but also neutrophils from other mammals release NETs when they are stimulated with *E. bovis* sporozoites, suggesting that NETosis is a conserved immune mechanism among animals.

It has been described that *E. arloingi* is also capable to induce *in vitro* NETosis in caprine neutrophils [93]. Like *E. bovis, E. arloingi* sporozoytes induce a rapid NETosis process that depends on ROS from NADPH oxidase; in the same manner, NETs entrapped and also reduced the infectivity of *E. arloingi* parasites over BUVEC cells, but the extruded DNA did not affect the viability of sporozoytes. NETosis was not a stage-specific mechanism since *E. arloingi* oocysts were also able to induce NET release.

The *in vivo* occurrence of *Eimeria*-induced NETosis has been recently documented [94]. In cross-sections from bovine and calf intestinal gut samples infected with *E. bovis* and *E. arloingi*, respectively, the presence of leukocytic infiltration mainly composed of neutrophils, and eosinophils and monocytes were detected. The immune cells were observed in close contact with oocytes, macrogamonts, and macromeronts. Interestingly, the presence of extracellular DNA co-localizing with histones and NE was also detected in this samples, indicating the occurrence of NETosis in infected tissues. It is to notice that the released DNA, which co-localized with anti-microbial proteins in the tissue samples, is principally attributed to neutrophils [17,54,95]; nevertheless, other leukocytes such as eosinophils, monocytes, and macrophages can also be able to drive ET formation [22,96,97]. For this reason, it is important to take care with data interpretation due to some of the principal NET hallmarks (e.g., histones, NE, or MPO) are not exclusive of neutrophils [98,99] (Figure 1D).

### Toxoplasma gondii

Toxoplasmosis is caused by *T. gondii*, an obligate intracellular protozoan pathogen from phylum Apicomplexa. In various places throughout the world, it has been shown that in some locations, up to 95% of populations have been infected with *Toxoplasma* [100] and the prevalence varies widely from 10 to 80% between countries [101]. Oocysts are produced during the sexual reproduction of the parasite exclusively in felines, which are the definitive host. After oocyst ingestion by intermediate hosts such as humans, other mammals, and birds, sporozoites are released and penetrate the intestinal epithelium developing into tachizoytes. Tachyzoites divide by endodyogeny inside any type of nucleated cells and disseminate throughout the organism. Tachizoytes can differentiate into bradyzoites producing tissue cysts and may remain throughout life in most hosts, predominantly in the brain or musculature. Tissue cysts are infective when they are ingested by intermediate hosts. After ingestion of these tissue cysts, they are ruptured in the digestive tract releasing bradyzoites. The bradyzoites will infect the intestinal epithelium developing into the tachyzoite stage for dissemination throughout the body [101,102].

Neutrophils appear to play an important role in the protection during the acute stage of the infection by *T. gondii*. It has been reported that mice infected with *T. gondii* died rapidly when neutrophils were depleted [103] and CXCR2^-/-^ mutant mice that showed a defective migration of neutrophils to the sites of infection had increased numbers of cysts [104]. In addition, neutrophils have an immunomodulatory role in this parasitosis [105]; evidence exist that different subsets of neutrophils are recruited at the infection sites to combat *T. gondii* pathogen [106].

It has been reported that in murine neutrophils NETosis was triggered by type I, II, and III strains of *T. gondii* and it did not require active parasitic invasion [95]. NETs caused damage in the plasma membrane of parasites and reduced their capacity to infect fibroblasts, indicating reduction in the parasite viability. Similar results were obtained *in vivo* using an intranasal infection mouse model. In lung sections, it was observed inflammatory infiltrate composed principally by neutrophils as seen from positive immunohistochemcal staining for MPO and from abundant dsDNA in bronchoalveolar lavage fluid (BLS) in control mice compared with neutrophil-depleted mice, suggesting that the detected DNA was from neutrophils. In addition, tachizoites recovered from BLS of neutrophil-depleted mice conserved their infectivity in fibroblast cultures; however, the infectivity of the tachizoites recovered from the control mice was reduced, indicating the impact of NETs on the control of toxoplasma infection.

Furthermore, *T. gondii* tachizoites induced NET release in a human promyelocytic leukemia cell line (HL-60) differentiated with dimethyl sulfoxide to neutrophil like-cells and in peripheral blood human neutrophils; in both cases, NETs entrapped tachizoite forms in presence of cytochalasin D, indicating that NETosis occurred independently of active parasitic invasion. It is important to mention that HL-60 cells present different responses depending on the stimuli used to their differentiation: all-trans retinoic acid-differentiated HL-60 cells released NETs only upon PMA stimulation, DMSO-differentiated HL-60 cells only upon A23187 calcium ionophore stimulation, while dimethylformamide-differentiated HL-60 cells formed NETs in response to either stimuli [107]. In this sense, *T. gondii*-induced NETosis can likely implicate a calcium influx taking into account the case of HL-60 cells differentiated with DMSO. The ERK1/2 MAPK signal pathway was involved in NETosis induced by *T. gondii* in human neutrophils since MEK inhibition decreased the extruded DNA levels; nevertheless, NETosis was not completely abolished, suggesting that other signaling pathways are involved in the process. It is worth to notice that recognition mediated by TLR was not necessary to trigger NET release by this parasite in contrast with *T. cruzi*-induced NETosis where TLR signaling was required [65]. These data confirm that different pathways drive NETosis depending upon the stimuli used as it has been previously observed [85].

### Neospora caninum

*N. caninum* is an apicomplexan parasite that can infect different mammalian hosts including cattle, dogs, horses, goats, sheep, deer, and others. This parasite is the principal cause of abortion in cattle in many countries [108].

*In vitro* NETosis induced by *N. caninum* tachyzoites has been described in neutrophils from dogs, bovines, and dolphins [109–111]. In all cases, extracellular DNA fibers were detected entrapping tachyzoites and co-localizing with NE, MPO, and histone H3, which confirms that these structures corresponded to NETs. Nevertheless, the mechanism implicated in NET release by *N. caninum* varies depending on the neutrophils origin. NETosis induced in canine neutrophils showed a time- and dose-dependent kinetics and NADPH oxidase, NE and MPO activities were required for it, whereas time and dose dependency was not observed in bovine neutrophils and the inhibition of NE and MPO enzymatic activities reduced NETs but the differences were not statistically significant. In this regard, while ERK1/2 and p38 MAPK activation as well as the SOCE led to NETosis in canine neutrophils stimulated with *N. caninum* tachyzoites, the inhibition of these signaling pathways did not cause a significant decrease in NET release bovine neutrophils. Furthermore, NETosis induced by *N. caninum* in bovine neutrophils was also independent of PAD4 activity and CD11b (Figure 1D).

A summary of the characteristics of the NETosis process triggered by the protozoan parasites is shown in Table 1.

**Table 1:** Summary of the characteristics of NETosis mechanisms induced by protozoan parasites

Parasite	Receptor implicated	ROS dependency		Enzymatic activity required			Signal pathway involved	Anti-parasitic effect	Reference
		NOX-2	Mitochondria	NE	MPO	PAD4			
*Leishmania donovani*	ND	(−)	ND	ND	ND	ND	ND	(+)^1,3^	[55]
*Leishmania amazonensis* classical NETosis	ND	(+)	(−)	(+)	(−)	(+)	PI3Kγ/ERK/PKC	(+)^3^	[56,57]
*Leishmania amazonensis* early/rapid NETosis	ND	(−)	ND	(+)	ND	(−)	PI3Kδ/Calcium	(+)^3^	[56,57]
*Trypanosoma cruzi*	TLR2/4	(+)	ND	ND	ND	(+)^2^	ND	(+)^3^	[65]
*Entamoeba histolytica*	ND	(−)	ND	(+)	ND	(−)	Raf/MERK/ERK	(−)	[75,83,84]
*Eimeria bovis*	CD11b	(+)	ND	(+)	(+)	ND	ERK1/2, p38 MAPK	(+)^4^	[90,91]
*Eimeria arloingi*	ND	(+)	ND	(+)	(−)	ND	ND	(+)^4^	[93]
*Toxoplasma gondii*	TLR independent	ND	ND	ND	ND	ND	ERK1/2	(+)^3^	[95]
*Neospora caninum*	ND	(+)	ND	(+)	(+)	(−)	ERK 1/2, p38 MAPK	(+)^5^	[109–111]

ND, Not determined.

1 LPG protects against anti-parasitic effect of NETs.

2 Determined by histone citrullination.

3 NETs caused death of parasites.

4 NETs reduced parasite infectivity.

5 NETs entrap parasites.

## Helminths parasites: an overview

It is estimated that helminths infect more than 2 billion people in developing countries causing high rates of morbidity mainly associated with anemia, malnourishment, and weakness of the host [112]. Helminth infections are usually acquired by the ingestion of water or food contaminated with fecal matter that contains helminth eggs, the ingestion of encysted larvae with meat, or by the penetration of *larva migrans* through the skin into different target organs.

The helminth infections are generally chronic due to the ability of these parasites to manipulate the immune response of the host through the secretion of immunomodulatory products. As with most infections, helminths initially induce in the host an innate immune response that is essential for the development and establishment of protective adaptive immunity. This adaptive immune response has been suggested to be of Th2-type. The cross-talk between innate and CD4+TH2 cells results in finely adjusted effector responses that control the spreading of the parasite. Although there are larvae of parasite helminths small enough to be phagocytosed by polymorphonuclear cells and macrophages, most of them are too large to be eliminated by this route. The invasion of host tissues by helminths usually leads to the development of granulomas aimed to encapsulate the parasite preventing its dissemination and to destroy it, therefore reducing the possibility of dispersion of antigens that promote inflammation and tissue damage. Macrophages and neutrophils are the earliest cells to be recruited within the granuloma or adjacent tissues followed, depending on the helminth parasite, by the infiltration of eosinophils, basophils, and mast cells (reviewed in [113]).

The evidence suggest variable role of the neutrophils in mediating resistance to helminths, which seems to depend on the context of the immune response and the parasite in question. Thus, it has been described that human neutrophils were able to kill the infective stage of the nematode *Strongyloides stercoralis* in a complement-dependent way when these cells were cultured together with macrophages [114]. Nevertheless, when neutrophils or macrophages were cultured separately with the parasite, they failed to kill *S. stercoralis* larvae. Noteworthy, in this study it was also demonstrated that soluble factors released by neutrophils and macrophages cross-activate them to kill the parasite, showing that they cooperate to establish a protective defense. Similar cooperation for optimal killing had also been reported for eosinophils [115]. A major role of neutrophils against the early stages of skin infection by the filaria *Litomosoides sigmodontis* has also been reported in a neutropenic murine model [116]. In this study, the anti-filarial activity of infiltrating neutrophils was associated with degranulation, oxidative burst, and NETs release effector mechanisms. In contrast, depletion of neutrophils in a murine model had no effect on the severity of tissue damage caused by the trematode *Schistosoma mansoni*, suggesting that these neutrophils have no protective role in this infection [117]. More recently, it was reported that the recruitment of neutrophils to the liver of mice by gamma-delta T cell subset Vγ2 T, which secrete IL-17A, aggravates the fibrosis caused by an infection with *S. japonicum*, and contributes to the pathology of the disease [118].

## Helminth parasites and NETs

Our understanding of the role of the NETs action against helminth parasites remains incipient. Few studies have been carried out compared with the work done with protozoa; however, some working groups have contributed to a better understanding of the role played by neutrophils in these diseases. Below, we provide the information available regarding the production of NETs induced by metazoan parasites.

### Strongyloides stercoralis

*S. stercoralis* is the etiological agent of strongyloidiasis, a chronic parasitic infection of dogs, cats, and primates including humans principally in the tropical and subtropical regions and, in less extent, in countries with temperate climates. According to WHO, an estimate of 30–100 million people are infected worldwide with *S. stercolaris*; precise data on prevalence are unknown in endemic countries [119,120].

Infection is acquired when filariform larvae from contaminated soil penetrate the human skin and migrate to the small intestine where they molt twice and become adult female worms. The females live in the epithelium of the small intestine and produce eggs by parthenogenesis. The rhabditiform larvae pass either to the stool or become infective filariform larvae, which can penetrate either the intestinal mucosa or the skin of the perianal area causing autoinfection [119,121].

**Figure 2:**
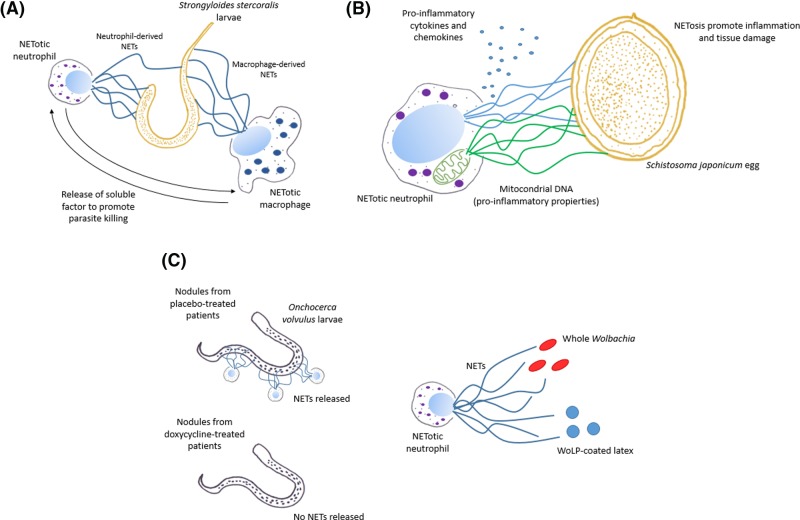
NETosis triggered by helminth parasites and its implications (**A**) *Strongyloides stercoralis* larvae trigger NETosis in human neutrophils and macrophages. Both cell types are unable to kill the larvae separately; however, together they kill efficiently this parasite due to the release of soluble factors that enhance the anti-parasitic effect of NETs. (**B**) *Schistosoma japonicum* eggs induce NET release in human neutrophils. During this NETosis process, mitochondrial DNA and pro-inflammatory cytokines and chemokines are released. These molecules promote an inflammatory response that causes tissue damage observed in the hepatic granuloma caused by this parasite. (**C**) In nodules excised from placebo-treated patients infected with *Onchocerca volvulus*, the presence of NETs in close contact with the parasite cuticle was detected; in contrast, NETs were not detected in doxycycline-treated patients, suggesting that the symbiotic bacteria *Wolbachia* was responsible for triggering NETosis. Confirming this theory, whole *Wolbachia* and latex beads coated with its lipopeptide (WoLP) triggered *in vitro* NET release in human neutrophils.

Human neutrophils co-cultured with viable and non-viable *S. stercoralis* larvae formed NETs *in vitro* and independently of complement [114]. As previously mentioned, neutrophils or macrophages separately cultured with *S. stercoralis* larvae did not kill worms; nevertheless, when both cells were cultured together with larvae, they killed 90% of parasites. Interestingly, this effect was reduced by the addition of DNase I, suggesting that the parasite killing was mediated by NETs. Furthermore, human macrophages were able to release ETs in response to *S. stercoralis*, suggesting that ETs released by macrophages could contribute to the killing activity. NETosis by *S. stercoralis* larvae, however, was also induced in murine neutrophils but not in murine macrophages. In this case, the anti-parasitic action of these cells was not mediated by NETs since the addition of DNase to the cultures did not reduce the killing efficiency of leukocytes. *S. stercoralis*-induced NETosis was detected *in vivo* inoculating L3 larvae in the peritoneal cavity of mice where it was observed increase of free DNA in peritoneal lavages in respect to the controls, and NETs were able to kill the larvae (Figure 2A).

### Schistosoma japonicum

Schistosomiasis is caused by different species of trematodes from genus *Schistosoma*. It is prevalent in tropical and subtropical areas, especially in poor communities. It is estimated that at least 91.4% of the persons requiring treatment for schistosomiasis live in Africa. Intestinal schistosomiasis is caused by *S. mansoni, S. japonicum, S. mekongi*, and *S. guineensis* species and the infection is acquired when infective cercariae penetrate the skin of the human host and shed their forked tail, becoming schistosomulae. The schistosomulae migrate through tissues and stages to their residence in the veins. In humans, the adult worms reside in different locations in the mesenteric venules. The females deposit eggs in the small venules of the portal and perivesical systems. The eggs are moved progressively toward the lumen of the intestine and are eliminated with feces. Some of the eggs, however, may also become lodged in the host’s intestine, liver, or other sites causing granulomas [122–124]. During a transcriptomic study of hepatic granulomas caused by *S. japonicum* and *S. mansoni* in a mouse model [125], the presence of neutrophil infiltrations in the core and periphery of *S. japonicum*-induced granulomas was detected. Noteworthy, NET-like structures were visualized surrounding the parasite eggs in contrast with the *S. mansoni* granuloma where less abundant neutrophils were observed only in the periphery and no NETs were visualized. *In vitro*, only *S. japonicum* eggs were able to trigger NET release. Furthermore, it has been described that whole soluble protein or excretory/secretory protein from *S. japonicum* eggs is able to induce NETs accompanied by the release of mitochondrial DNA and pro-inflammatory cytokines and chemokines [126]. In this regard, it is probable that NET contributes to the damage associated with granuloma by propitiating inflammation since mitochondrial DNA released during NETosis possess pro-inflammatory properties and it has been linked to pathology associated with severe diseases [127,128]. Furthermore, NETs did not affect the viability of *S. japonicum* eggs entrapped, suggesting the non-protective role of NETosis in this parasitic disease (Figure 2B).

### Haemonchus contortus

*H. contortus* is a highly pathogenic helminth, primarily of small ruminants, with a global distribution. The prevalence of *H. contortus* is particularly high in the tropical climatic zones of both hemispheres; nevertheless, *H. contortus* presents adaptability over a wide range of environments. Ruminants are infected when they ingest the L3 infective larvae stage, which pass through the first three stomachs establishing finally in abomasum. L3 larvae shed their cuticles and burrow into the internal layer, where they develop into L4 larvae stage. The L4 larvae then molt and develop into adult form. The adult male and female parasites feed on blood and may remove up to 30 μl of blood per day, rapidly causing anemia and subsequent death [129,130].

*H. contortus* L3 larvae induced NETosis in bovine neutrophils [131]. Three morphological types of NETs were observed by scanning electron microscopy and immunofluorescence during the interaction of the neutrophils with the larvae according to: diffuse NETs characterized by their globular and compact form, spread NETs observed as smooth elongated web-like structures composed by thin fibers, and at last, aggregated NETs denoted by large clusters of NET-like structures with a ‘ball of yarn’-like clumpy massive appearance involving a high number of neutrophils. Only the aggregated NETs showed the ability to immobilize the larvae, but like with other parasites, NETs did not kill larvae. NETosis was triggered independently of parasite viability as viable and heat-inactivated larvae indistinctly induced NET release. Neutrophils were able to extrude DNA in acidic conditions, indicating that NETosis can well take place *in vivo* in the abomasum that presents low pH values. Like in other cases, the process depended on NADPH oxidase, MPO, and NE activities.

### Nyppostrongylus brasiliensis

*N. brasiliensis* is a nematode parasite that infects rodents including *Rattus norvegicus, R. rattus*, and *Mus musculus*. After percutaneous infection, the L3 larvae stage migrates to the lungs. Here they develop into L4 larvae stage and migrate via the trachea and esophagus to the small intestine. The larvae grow and transform into adult worms. The adults mature and female worms produce eggs. Egg production continues for about a week and then most of the worms are expelled from the rodent [132]. During inoculation of non-viable *N. brasiliensis* L3 larvae in ear skin of mice, inflammatory neutrophils and monocytes were rapidly recruited followed by the appearance of NET-like structures characterized by the presence of extracellular DNA co-localizable with NE, MPO, and citrullinated histone H3 [133]. These structures were also produced in both TLR2 KO and TLR4 KO mice, suggesting that NETosis triggered by *N. brasiliensis* did not require TLR signaling.

## Filarial nematodes

Filarial diseases are a prevalent public health problem. According to WHO, an estimate of 120 million people in 73 countries from tropical and subtropical areas are infected with lymphatic filariasis [134,135]. Approximately, 90% of filarial infections are caused by *Wuchereria bancrofti* and the remainder by *Brugia* spp. Humans are the exclusive host of infection with *W. bancrofti* whereas certain strains of *Brugia malayi* can also infect some animal species. River blindness caused by *Onchocerca volvulus* is found in some foci in Latin America but more than 99% of infected people live in 31 countries in sub-Saharan Africa [135]. The life cycle of filarial nematodes involves uptake of the first-stage larvae (microfilariae, Mf) by a hematophagous arthropod followed, after two moults in the vector, by transmission of the L3 larvae stage to a new vertebrate host, where two further moults later, the nematodes mature as dioecious adults [136]. Noteworthy, filarial nematode species *Brugia* spp., *W. bancrofti*, and *O. volvulus* usually are infected by *Wolbachia*, a genus of Gram-negative intracellular bacteria belonging to the order Rickettsiales of the family Anaplasmataceae. These bacteria only infect invertebrate organisms and are naturally found in several nematodes [137,138]*.* The nature of the symbiosis between *Wolbachia* and nematodes has been considered mutualistic since antibiotic chemotherapy in various Onchocercid species has demonstrated that depletion of *Wolbachia* is associated with stunting, sterilization, and death of adult worms [139]. In addition, *Wolbachia* is associated with inflammatory process present in filarial diseases.

The presence of NETs containing NE, MPO, and citrullinated histones was detected in nodules excised from placebo-treated patients infected with *O. volvulus* nematodes, which were positive for *Wolbachia*. NETs were visualized by immunofluorescence in close contact with the parasite cuticle [140]. In contrast, NETs were not detected in doxycycline-treated patients and nematodes were negatives for *Wolbachia*, suggesting that the symbiotic bacteria was responsible for triggering NETosis. In this sense, whole *Wolbachia* and its lipopeptide (WoLP) bound to latex beads were able to induce NET release in human neutrophils while soluble WoLP failed to do it. This mechanism was triggered via TLR6 since neutrophils from mutant mice lacking TLR6 were unable to form NETs in the presence of whole *Wolbachia* and WoLP-coated latex beads (Figure 2C).

*B. malayi* microfilaria induced NETosis in human neutrophils, and in these structures the presence of MPO, NE, and citrullinated histone H4 was detected [141]. In the case of *B. malayi* microfilaria, NET release was dependent on ROS since NADPH oxidase inhibition led to a decrease in the extruded DNA. Interestingly, addition of autologous serum to the cultures blocked NETosis but this action cannot be attributed to the complement as the heat-treated serum also inhibited NETosis. NET release promoted the attachment of neutrophils to *B. malayi* microfilaria; nevertheless, NETs do not apparently participate in parasite killing because the addition of DNase I to the cultures did not reduce the killing efficiency of neutrophils.

*Litomosoides sigmodontis* is a filarial nematode that infects rodents and is commonly used as a model to study filarial diseases. NETosis was detected in murine neutrophils stimulated with *L. sigmodontis* accompanied by an increase in ROS generation and the release of MPO from cells. *L. sigmodentis*-induced NETosis was observed *in vivo* in skin from mice inoculated with the parasite as extracellular DNA co-localized with NE and MPO by immunofluorescence.

## Concluding remarks

The study of NETs has advanced by leaps and bounds since they were first described only 14 years ago. Many of the studies have focused on the identification of the stimuli that induce NETs, and on the neutrophil signaling pathways involved. This is particularly true in the case of parasites, some of which trigger NETs by the classical routes, i.e. ROS and PAD4-dependent routes, while others, such as the amoeba, do it in a non-classical way. The panorama seems to be more complicated by the results obtained in the studies of NETs formation by helminth parasites and the identification of the *Wolbachia* symbiont of the filarial *O. volvulus* as one of the stimuli of NETs associated with this parasite. This suggests the possibility that the microbiota of helminths, as well as viruses and bacteria carried by protozoan parasites, would be able to influence the pathways of the formation of traps in the neutrophils and, perhaps, their outcome. This is of particular importance if we consider that it has been suggested that large particles such as parasites would induce the formation of NETs whereas small particles, such as bacteria and viruses, would induce phagocytosis [39,40]. Since both viruses and bacteria can induce NETs formation under certain conditions, it is likely that other signals derived from the particle (e.g., pathogenicity and/or virulence signals) and the condition of the neutrophil (primed or not, stressed or not, in circulation or in tissue) are also equal or more important for the outcome under physiological conditions [41,42].

The exact role that NETs play during parasitic infections remains unknown, being an area of intense study at present. Most *in vitro* evidence indicate that NETs can trap protozoa, and in some cases, such as promastigotes of *Leishmania* or tachyzoites of toxoplasma, to kill them. In contrast, in cases such as *E. histolytica* trophozoites, the NETs do not seem to affect the viability and infectivity of the parasite. Something similar has been observed in the case of helminth parasites, where NETs trap and kill the larvae of some of these parasites, but they do not seem to affect the viability of adult worms.

Even less known is the role of NETs during the invasion of host tissues by parasites. Advances in this regard have been hampered by the difficulty in the sure identification of NETs in infected tissues and by the absence of specific inhibitors of *in vivo* NETs formation. The possibility that NETs participate in the pathology rather than in the defense of the host against parasites also remains latent. In host tissues infected with parasites, the NETs may not spread enough to trap them and, instead, the traps may exacerbate the local inflammatory response by activating the complement and coagulation systems, contributing thus to tissue damage [142]. This could help explain the large tissue damage associated with some parasitic infections, such as amoebiasis and schistosomiasis.

Finally, the elucidation of the role of NETs and the understanding of the cellular mechanisms behind their regulation could contribute to the development of more efficient strategies of the control of parasitic infections and the amelioration of the damage caused by them in humans and animals of veterinary importance.

## Abbreviations

BLS: bronchoalveolar lavage fluid
ET: extracellular trap
LPG: lipopetidoglycan
MPO: myeloperoxidase
NE: neutrophil elastase
NET: neutrophil extracellular trap
PAD4: peptidyl arginine deiminase 4
PKC: protein kinase C
PMA: phorbol miristate acetate
ROS: reactive oxygen species
WHO: World Health Organization
